# Peritoneal Keratin Granulomatosis Associated with Endometrioid Adenocarcinoma of the Uterine Corpus in a Woman with Polycystic Ovaries: A Potential Pitfall—A Case Report and Review of the Literature

**DOI:** 10.1155/2017/1863215

**Published:** 2017-04-19

**Authors:** Helen J. Trihia, Maria Papazian, Natasa Novkovic, John Provatas, Sotiria Tsangouri, Dimitrios C. Papatheodorou

**Affiliations:** ^1^Department of Pathology, “Metaxas” Cancer Hospital, 18537 Piraeus, Greece; ^2^Department of Cytology, “Metaxas” Cancer Hospital, 18537 Piraeus, Greece; ^3^Department of Gynaecology, “Metaxas” Cancer Hospital, 18537 Piraeus, Greece

## Abstract

Peritoneal keratin granulomatosis is a rare condition included under granulomatous lesions of the peritoneum. It can be secondary to neoplasms of the female genital tract and can mimic carcinomatosis intraoperatively. A case of a 40-year-old woman with a history of polycystic ovaries and a chief complaint of vaginal bleeding is presented. She was diagnosed with endometrioid adenocarcinoma with squamous differentiation in endometrial curettings. Intraoperatively, many peritoneal nodules were found, interpreted as peritoneal carcinomatosis. The woman underwent a total abdominal hysterectomy with bilateral salpingo-oophorectomy, omentectomy, bilateral pelvic lymphadenectomy, and appendicectomy. Multiple biopsies were taken, as well as peritoneal washings. Microscopic examination revealed multiple keratin granulomas on the serosal surface of the ovaries, fallopian tubes, appendix, and omentum. Lymph node metastasis was not found. Peritoneal keratin granulomas (PKGs) have been reported in cases of endometrioid adenocarcinoma with squamous differentiation of the uterine corpus, ovary, and atypical adenomyoma. It should be noted that the prognosis of cases of peritoneal keratin granulomas without viable tumor cells is favourable and that the histologic examination is essential for its diagnosis. We report a case of PKG in a patient with endometrial carcinoma with squamous differentiation, being the first in a woman with polycystic ovaries.

## 1. Introduction

Peritoneal keratin granuloma is a rare lesion included among reactive tumor-like lesions of the peritoneum. It can be secondary to endometrioid adenocarcinoma with squamous differentiation of the endometrium and ovary and atypical polypoid adenomyoma of the endometrium and in association with ruptured dermoid cysts. The prognostic significance of these lesions is unknown and it seems to have no interference with prognosis, when no viable tumor cells are detected. Here we describe a case of an endometrioid adenocarcinoma of the endometrium, in a woman with polycystic ovaries in which diffuse peritoneal keratin granulomas were found with no viable tumor implants which intraoperatively were misinterpreted as diffuse carcinomatosis.

## 2. Case Presentation

A 40-year-old woman with a body mass index (BMI) of 37 and a past medical history of polycystic ovary syndrome, presented to her gynaecologist complaining of irregular vaginal bleeding. Her menarche was at the age of 16 and her menstrual cycle was infrequent and irregular. Endometrial biopsies (D&C) have been examined at the age of 33 and 38 years. At the age of 38, she was diagnosed with atypical adenomatous hyperplasia of the endometrium and she was put on progestagen therapy. A few months later, she experienced a new episode of irregular vaginal bleeding and after an additional D&C she was diagnosed with endometrioid adenocarcinoma of the endometrium. As a routine pre-op check, tumor markers were requested. Her serum CA125 and serum CA19.9 were elevated to 69.00 U/ml (normal < 35.00 U/ml) and 91.60 U/ml (normal < 35.00 U/ml), respectively. The magnetic resonance imaging (MRI) of the lower abdomen revealed invasion of more than 50% of the myometrium and of the uppermost uterine cervical stroma. Blurring of the sigmoid fat and prominent inguinal, para-aortic and mesenteric lymph nodes were also described with a maximum lymph node diameter of 1.5 cm. Total abdominal hysterectomy, bilateral salpingo-oophorectomy, bilateral pelvic lymph node dissection, omentectomy, and appendicectomy were performed. Intraoperative peritoneal washings were also carried out. Multiple peritoneal nodules, <0.5 cm in diameter, suspicious of disseminated carcinomatosis, were found during surgery in the pouch of Douglas, over loops of small bowel, and in the mesentery of the small bowel. Multiple biopsies were taken. Due to increased BMI, para-aortic lymphadenectomy was not performed. No frozen section was requested because it was appreciated that a positive result would not affect the overall surgical management.

**Figure 1:**
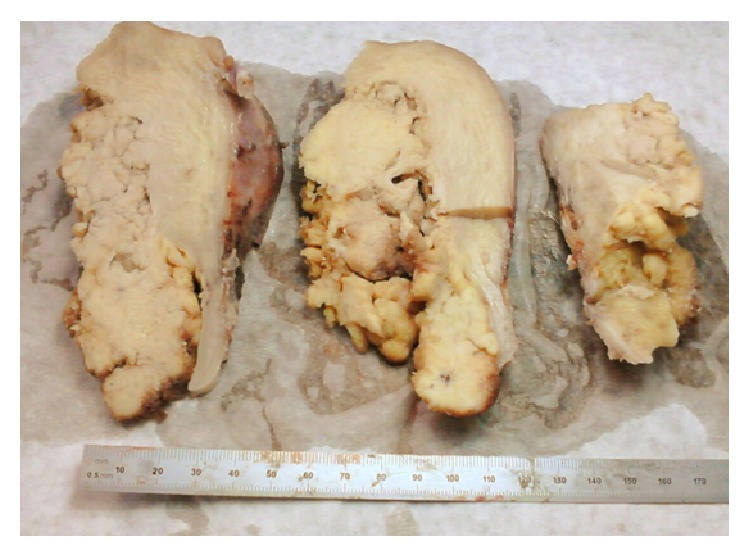
Macroscopic appearance of a cross-sectioned uterus filled with a polypoid papillary mass, extending into the uterine cervix.

Grossly, the uterine corpus, including both cornua, was filled with a polypoid papillary mass, measuring 11, 5 × 5, 5 cm, extending into the uterine cervix (Figure 1). Both ovaries were enlarged with multiple peripherally located follicular cysts and dense peripheral stroma, consistent with the clinical history of polycystic ovaries.

**Figure 2:**
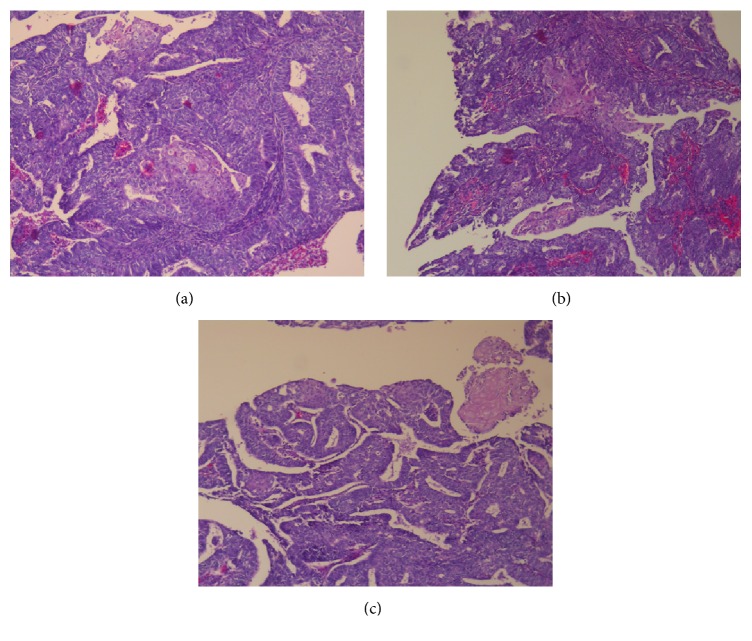
Microscopic appearance of endometrioid carcinoma with foci of squamous differentiation.

**Figure 3:**
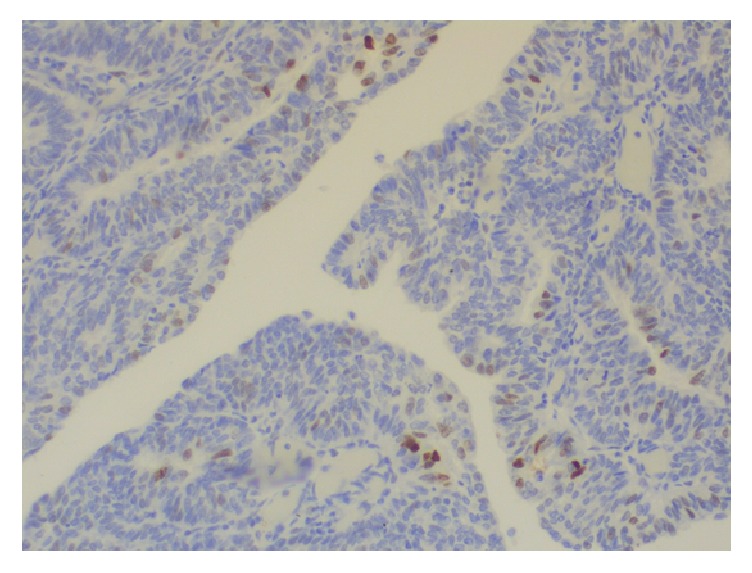
Immunostain: positive expression of p53 in endometrial carcinoma.

**Figure 4:**
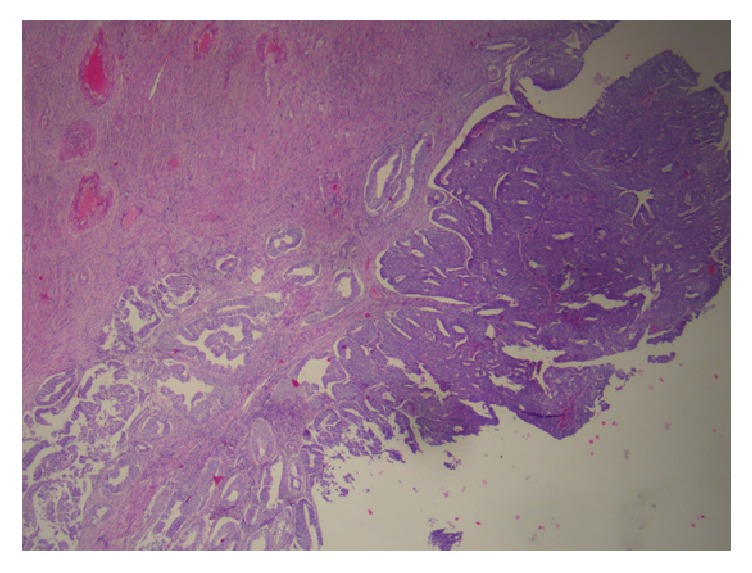
H&E stain: superficial invasion of the uterine cervix by the endometrial carcinoma.

**Figure 5:**
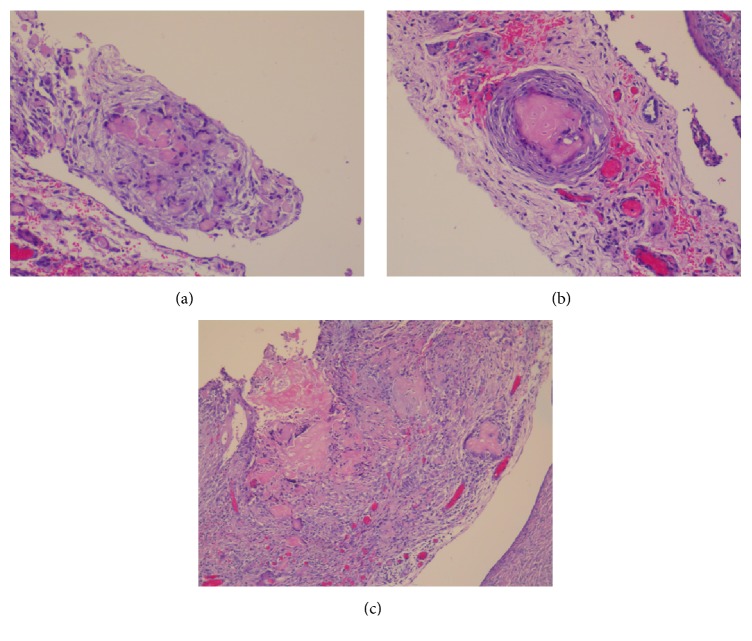
H&E stain: microscopic appearances of multiple keratin granulomas, composed of ghost squamous cells surrounded by foreign body giant cells, found on the serosal surface of bilateral ovaries, fallopian tubes, the appendix, and omentum.

**Figure 6:**
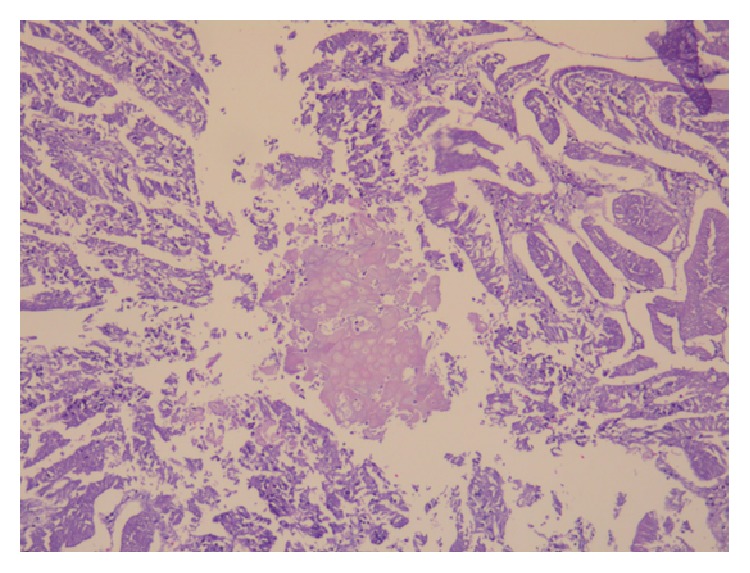
H&E stain: degenerate squamous cells found in superficial areas of the uterine tumor.

**Figure 7:**
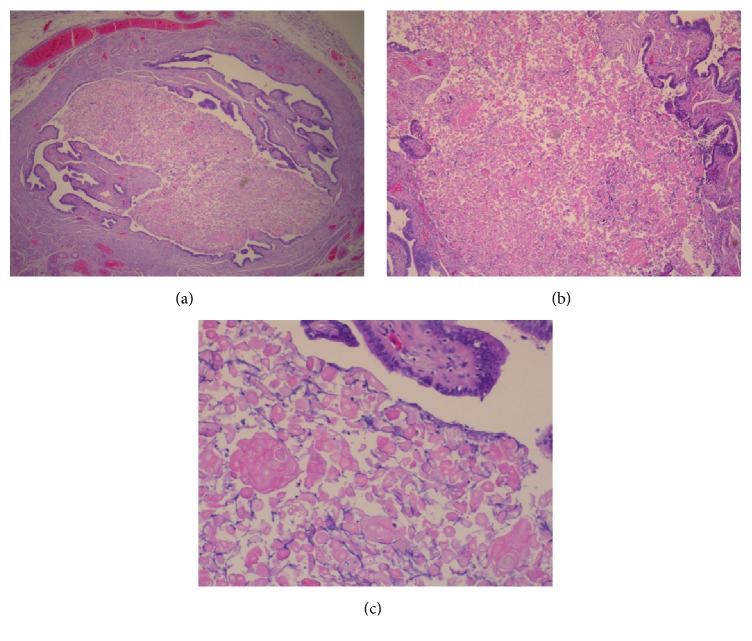
H&E stain: similar to Figure 6, degenerate squamous cells are filling and distending the lumen of the fallopian tubes (in various magnifications).

Histologically, the tumor of the uterine corpus was a superficially invasive, moderately differentiated, tubulopapillary adenocarcinoma of the endometrium, of endometrioid type with multiple foci of squamous differentiation (Figures 2(a)–2(c)). Immunohistochemically, there was positive expression of hormone receptors and p53 (Figure 3). The tumor was extending superficially to the uterine cervix (Figure 4). All 18 pelvic lymph nodes were unremarkable. In addition, on the serosal surface of bilateral ovaries, fallopian tubes, and the appendix, multiple microscopic granulomas were found, composed of amorphous irregularly laminated eosinophilic deposits of keratin, associated with ghost squamous cells and surrounded by foreign body giant cells (Figures 5(a)–5(c)). There were also reactive mesothelial cells close to keratin granulomas. In retrospect, similar degenerate squamous cells were found in extensive, mainly superficial areas of the uterine tumor (Figure 6) as well as filling and distending the lumen of the fallopian tubes, bilaterally (Figures 7(a)–7(c)).

Intraoperative peritoneal washings showed scattered mesothelial cells, occasional clusters of atypical cells of mesothelial origin, rare anucleate squames, and an occasional keratin granuloma. Overall, the endometrial carcinoma was of UICC/FIGO stage II.

## 3. Discussion 

Peritoneal keratin granuloma is a rare lesion included under granulomatous lesions of the peritoneum [1]. Such peritoneal reaction can be infectious or noninfectious in aetiology [1]. The noninfectious type can be secondary to neoplasms of the female genital tract, like endometrioid adenocarcinoma with squamous differentiation of the endometrium and ovary and atypical polypoid adenomyoma of the endometrium or seen in association with ruptured dermoid cysts. They are also found in ruptured ovarian teratoma and nonneoplastic conditions, such as spilled amniotic fluid, or in intraperitoneal renal dialysis-associated peritoneal squamous metaplasia [1].

Peritoneal keratin granulomas refer to the finding of nests of keratinized anucleate squamous cells surrounded by a foreign body type giant cell reaction, either on the peritoneal surface or within subperitoneal connective tissue. These so-called keratin granulomas do not contain any glandular epithelium. The typical histological appearances in previously reported cases were similar to ours.

**Figure 8:**
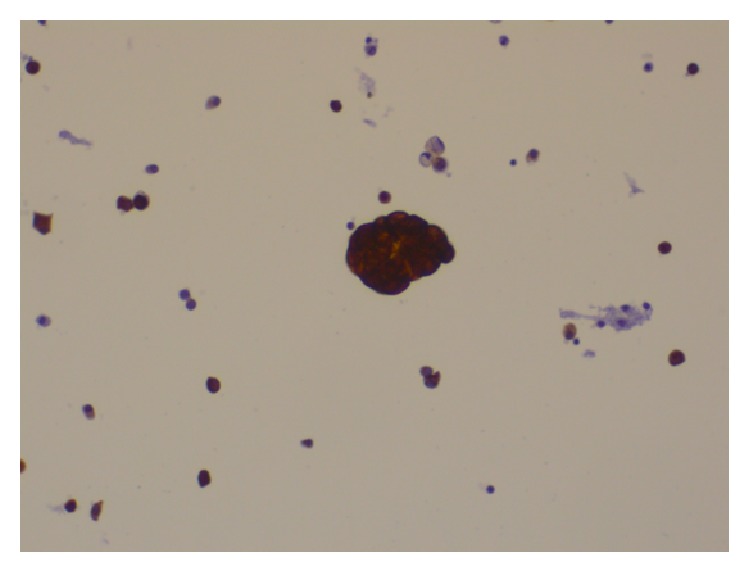
Immunostain for calretinin: atypical cell cluster in peritoneal washings of mesothelial origin.

Spontaneous reflux of exfoliated necrotic squamous metaplastic cells or keratin from the squamous element of the endometrial tumor to the peritoneum or its retropulsion through the tubal lumina due to endometrial sampling has been postulated as the pathogenetic mechanism of the 27 cases [2–7] of PKG associated with an endometrial adenocarcinoma with squamous differentiation [2–5]. The above induce a foreign body granulomatous reaction [3] and include frequent association with cervical stenosis, corneal location of the primary tumor, presence of keratin clumps within the lumen of the tube, and superficial location of squamous necrotic cells in the endometrial carcinoma [4]. In our case, all the above-mentioned requirements were met. The uterine cavity was filled with a tumor which was causing distention of the corneal part of the fallopian tubes and there was extensive squamous differentiation of the endometrioid adenocarcinoma, in about 1/20 of the tumor, which was more pronounced in superficial areas, where extensive degeneration and necrosis of the endometrial carcinoma were present (Figure 8). The tubes were massively distended and filled with numerous anucleate squames, which obviously spread to the peritoneum, leading to a florid granulomatous peritoneal reaction to keratin.

**Figure 9:**
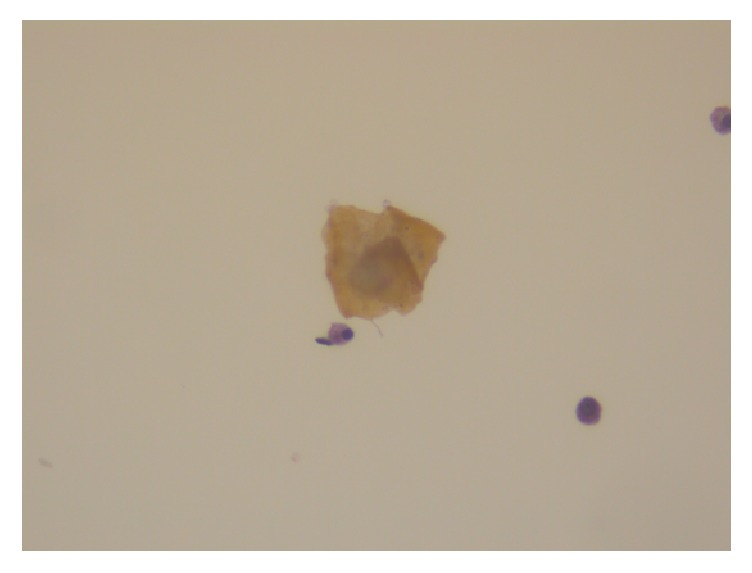
Pap stain: scattered anucleate squame in peritoneal washings.

**Figure 10:**
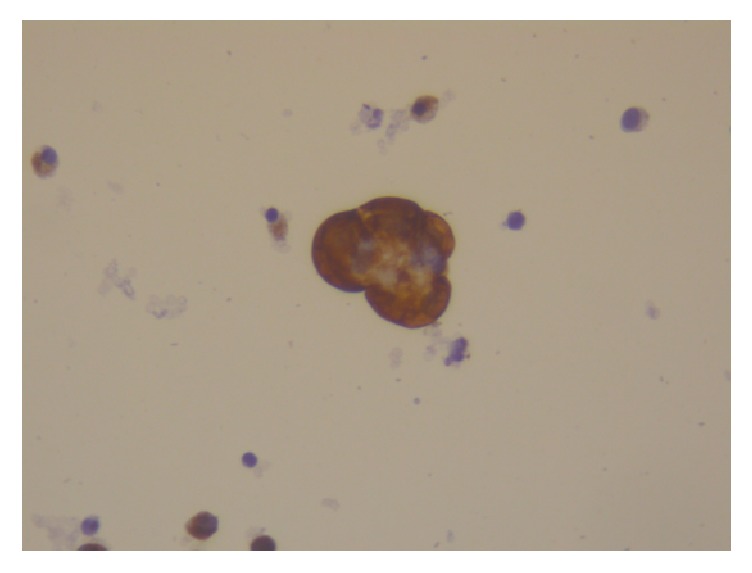
Immunostain for Ker5/6: keratin granuloma with positive expression in peritoneal washings.

The commonly reported process of keratinization in endometrial carcinomas could be influenced by irradiation, surgical trauma, partial removal of the tumoral mass, hormonal factors, infection, or transfusion, but the aetiology is usually unknown [4]. Sometimes the tumor cells may undergo keratinization after entering into the peritoneal cavity [4]. These may be visible to the surgeon and mimic peritoneal carcinomatosis macroscopically, as it was the case with our surgeons. As long as no glandular component is identified histologically, keratin granulomas should not be considered tumor spread and should not result in upstaging. In such cases, the areas should be thoroughly sampled by the gynaecologist and carefully examined microscopically by the pathologist to exclude the presence of viable tumor cells. Furthermore, reactive mesothelial hyperplasia near the keratin granulomas may occur. Peritoneal washings no longer contribute to endometrial cancer staging; nevertheless, they continue to be performed by the clinicians, even though a positive report may carry a risk of overdiagnosis, as it may be difficult to distinguish between reactive mesothelial and tumor cells. There is only one report on the cytohistological correlation of PKGs [8]. In our case, atypical cells in the peritoneal washings were immunoreactive for calretinin (Figure 8), indicative of mesothelial origin. There were also scattered anucleate orangeophilic squames (Figure 9), which could not have been of cutaneous origin, as the cytological sampling technique was not transcutaneous (paracentesis) and an occasional keratin granuloma, positive for ker5/6 (Figure 10). Cytological evidence of keratin in peritoneal washings does not infer a diagnosis of metastatic carcinoma, but careful scrutiny has to be done, to exclude the presence of malignant cells.

After revision of the literature, only 33 similar cases had been reported till 2012.

Tripathy et al. (2010) [7] and Montes et al. (1961) [9] were the first to describe a case of well-differentiated adenocarcinoma of the uterine corpus in which so-called “pigmented nodules” composed of foreign body keratin granulomas were identified on and below the serosal surface of the uterus and the proximal end of the fallopian tube. These authors suggested that squamous metaplasia and keratinization of endometriotic epithelium might lead to the formation of granulomas.

Chen (1978) [2] described five cases of uterine “adenoacanthoma” with peritoneal foreign body granulomatous reaction to keratin. These authors postulated that the pathway of entrance of keratin into the peritoneal cavity to be spontaneous reflux from the endometrial tumor, including a frequent association with cervical stenosis, a corneal location of the primary tumor leading to transtubal spreading. William et al. (1984) [10] and Wotherspoon et al. (1989) [5] reported two additional cases, associated with an “adenosquamous carcinoma” and an “adenoacanthoma” of the uterus, respectively. Kim and Scully (1990) [4] reported 22 cases of peritoneal keratin granulomas with carcinomas of endometrium and ovary and atypical polypoid adenomyoma of the endometrium, constituting the largest review of cases published in the literature. It was the first time that such peritoneal lesions were described to be related to endometrioid adenocarcinoma of the ovary (five cases). Wu et al. (2006) [11] described the other case of peritoneal keratin granuloma in association with ovarian adenocarcinoma. It was suggested by Kim and Scully (1990) [4] that tearing of the capsule of the tumor or malignant cell penetrating the ovarian surface was the way of cells in entering the peritoneal cavity. The last two published cases, by Van der Horst and Evans (2008) [6], refer to carcinomas of the endometrium also. A case with twelve-year follow-up of an endometrioid adenocarcinoma of the endometrium with disseminated peritoneal keratin granulomas and viable tumor implants was also reported in 2012 [12]. Interpreted as disseminated disease leading to a palliative approach with only brachytherapy and hormonal therapy, the outstanding survival could suggest no adverse effect on the prognosis of such peritoneal lesions even with viable tumor implants.

The prognostic significance of keratin peritoneal granulomas with or without viable tumor implants is difficult to assess because of the small number of cases in the literature. Lack of or short follow-up in some cases and postoperative radiotherapy, chemotherapy, or both, which might have influenced the natural course of any postoperative residual peritoneal lesions, makes it more difficult to interpret the real prognostic significance of these lesions. Some authors suggest that they have no prognostic significance when no viable cells are found in the granulomas.

The combination of contrast-enhanced T1-weighted and diffusion-weighted magnetic resonance is mentioned to be helpful for the preoperative differential diagnosis [13].

In the current study, we document a very rare case involving a patient with polycystic ovaries syndrome, who presented with a huge endometrial tumor which filled the uterus and protruded through the cervical os. The tumor was an endometrioid adenocarcinoma of the endometrium which was accompanied by multiple peritoneal keratin granulomas attributed to the squamous element of the tumor, transpassing the lumen of the fallopian tubes and eliciting a giant cell reaction. Our findings are in concordance with Chen et al.'s [3] and Wotherspoon et al.'s [5] proposed pathogenetic mechanism of spontaneous reflux of keratinized squamous cells through the lumen of the fallopian tubes into the peritoneal cavity in tumors associated with cervical stenosis or a corneal location. For the first time, we confirm microscopically the proposed pathogenetic mechanism of PKG formation. The fallopian tubes in our case were distended and filled with anucleate squames originated from the squamous metaplastic element of the endometrial adenocarcinoma.

Because peritoneal granulomatosis can resemble disseminated carcinomatosis macroscopically, the knowledge of this rare entity is essential to avoid upstaging of the patient. Our patient underwent brachytherapy and whole irradiation and is well after fifteen months of follow-up.

Furthermore, the findings of scattered anucleate squames and keratin granuloma in the peritoneal washings constitute the second cytohistologic reference of PKGs [8].
